# Near real-time adaptive isotropic and anisotropic image-to-mesh conversion for cerebral aneurysm simulations

**DOI:** 10.1007/s00366-026-02287-4

**Published:** 2026-02-17

**Authors:** Kevin Garner, Chander Sadasivan, Nikos Chrisochoides

**Affiliations:** 1 Department of Computer Science, Center for Real-time Computing, Old Dominion University, Norfolk, VA USA; 2 Department of Neurological Surgery, Cerebrovascular Center for Research, Stony Brook University, Stony Brook, NY USA

**Keywords:** Image-to-mesh conversion, Mesh generation, High-performance computing, Aneurysms

## Abstract

This paper presents two performance optimization techniques for a mesh adaptation method that is designed to help streamline the discretization of complex vascular geometries within the numerical modeling process. This method is integrated into a pipeline with an image-to-mesh conversion tool to generate adaptive anisotropic meshes from segmented medical images. The pipeline is shown to satisfy quality, fidelity, smoothness, and robustness requirements while providing near real-time performance for medical image-to-mesh conversion. Tested with two brain aneurysm cases and utilizing up to 96 CPU cores within a single, multicore node on Purdue University’s Anvil supercomputer, the parallel adaptive anisotropic meshing method utilizes a hierarchical load balancing model (designed for large, cc-NUMA shared memory architectures) and contains an optimized local reconnection operation that performs three times faster than its original implementation from previous studies. While utilizing a new user-defined sizing function, we also show an adaptive isotropic method that generates meshes with good quality and fidelity of up to approximately 50 million elements in less than a minute while the adaptive anisotropic method is shown to generate approximately the same number of elements in about 5 min.

## Introduction

Brain aneurysms are abnormal focal dilations of intracranial arteries which when left untreated may rupture, resulting in fatal outcomes for patients. High resolution Computational Fluid Dynamics (CFD) simulations provide the opportunity to study hemodynamics in arteries, which can assist clinicians in determining an aneurysm’s potential to rupture and possible methods of treatment (such as the design of neurovascular stents) [1, 2]. Stents used to treat brain aneurysms have wire sizes that can be as small as 25–30 microns with aneurysm sizes ranging from millimeters to centimeters. CFD studies on the effect of such devices (including overlapping devices) on intraneurysmal hemodynamics involve generation of volumetric meshes with elements at multiple sizes [3]. Processing patient-specific medical imaging data of cerebral aneurysms involves the discretization of these complex bodies in the form of smoothed free-form anisotropic body-conforming meshes. Mesh generation methods utilized in commercially available products work well when processing patient-specific cerebral aneurysm geometries; however, these methods do not always satisfy the time constraints of high resolution CFD simulations. These medical simulations can be classified into two categories—interactive and predictive. Interactive simulations allow surgical residents to practice their skills within a virtual environment and require real-time computations to provide high physics fidelity. Predictive simulations predict and optimize the outcome of an intervention using patient-specific pre-operative image data. Given their impact on a patient’s treatment and associated healthcare costs, both types of simulations are time-sensitive (and are impacted by a mesher’s real-time performance capability).

This paper presents two performance optimization techniques that are applied to a multicore cc-NUMA-based anisotropic mesh adaptation method known as CDT3D, which in turn is used to help streamline the discretization of complex vascular geometries within the numerical modeling process. Several requirements should be addressed with regards to medical image-to-mesh (I2M) conversion: robustness, fidelity, quality, smoothness, and real-time performance. Robustness concerns the software’s ability to process different input types such as CAD models (for example, of stents) and patient-specific medical images. Utilizing both is essential when running a medical simulation meant to aid in determining potential methods of treatment [4]. Fidelity measures the degree to which the mesh surface aligns with an image boundary. Quality (determined by the shape and size of mesh elements) and smoothness affect the accuracy of solutions for finite element solvers [5]. Smoothness refers to the geometric continuity of the mesh surface, characterized by $C^n$, where n represents the degree of continuity [6]. For example, $C^0$ represents a surface that is continuous with no gaps or holes but may contain sharp corners or edges. A surface of $C^1$ is tangent-continuous, lacking sharp corners. Smoothness also allows a mesh to reflect a certain degree of visual reality (important for interactive simulations [7]). Real-time performance is relative to the simulation in which the mesh generation application is utilized (what is considered “fast" in one simulation workflow might be slow in another). For example, the sizes of meshes utilized in some cerebral aneurysm simulations can vary from several thousand [8] to 200 million elements [9]. If a mesh generation application generates 10 thousand elements per second, it’d take 10 s to generate 100 thousand elements (which may be considered “fast" or real-time). When generating 200 million elements, that application would take about 5.5 h (which would no longer be considered real-time during an aneurysm treatment procedure). Ultimately, we define real-time as the capability to generate smooth meshes of high quality and fidelity typically in minutes or preferably in seconds so that the mesher can be integrated into time-critical clinical workflows without serving as a computational bottleneck in those workflows. Each of these I2M requirements are independently challenging problems. We integrate CDT3D into a pipeline of software tools that addresses the robustness, fidelity, quality, and (to a degree) smoothness requirements (providing a smoothness of $C^0$). The I2M pipeline involves three steps: (1) utilize a method known as CBC3D [10] to discretize a segmented image using a Body-Centered Cubic lattice of high-quality tetrahedra and deform the generated mesh surfaces to their corresponding tissue boundaries to improve fidelity while maintaining quality, (2) construct an anisotropic metric tensor field, and (3) utilize CDT3D [11] to generate an anisotropic mesh volume using the metric tensor field and CBC3D isotropic mesh as a background mesh. To help satisfy real-time requirements, CDT3D utilizes an optimized local reconnection operation and leverages a hierarchical model to balance workloads between threads.

We compare this optimized CDT3D method to a Delaunay-based image-to-mesh conversion software known as PODM (also called PI2M in [12]), where we’ve extended PODM’s functionality to adapt volume elements to now utilize a user-defined sizing function. PODM generates isotropic meshes from segmented images while satisfying fidelity, quality, and real-time requirements. We evaluate these methods using two aneurysm cases (a carotid cavernous aneurysm and middle cerebral artery bifurcation aneurysm) obtained from the Cerebrovascular Center for Research at Stony Brook University under Institutional Review Board approval. We show that both the anisotropic pipeline and isotropic PODM method meet the aforementioned I2M conversion requirements. Additionally, when utilizing 96 CPU cores on a single, multicore node on Purdue University’s Anvil supercomputer [13], the adaptive isotropic PODM method generates about 50 million elements in less than a minute while the adaptive anisotropic CDT3D method generates approximately the same amount in about 5 min. Given that CDT3D also exhibited excellent performance and accurately captured features of underlying aerospace simulations when coupled with a solver (also processing CAD data to perform adaptation [11]), the end goal is to utilize this method within a vascular flow simulation involving stents. This is however outside the scope of this paper and we first gauge the feasibility of utilizing the methods to help satisfy the aforementioned image-to-mesh conversion requirements (especially real-time performance capabilities given the below contributions).

Ultimately, this paper contributes the following:

1. a hierarchical load balancing model (designed to target large cc-NUMA shared memory architectures) for speculative execution applied to an adaptive anisotropic mesh generation method that only utilized 40 CPU cores for aerospace cases in earlier studies [11, 14] and is now tested up to 96 CPU cores for medical cases and
2. an optimized local reconnection algorithm for anisotropic adaptation, that when compared to its implementation in earlier studies [11, 14], is now three times faster.

Section 2 presents an overview of related work. Section 3 describes the above contributions in detail. Section 4 presents our evaluation of the methods while section 5 provides a further analysis/discussion. Section 6 discusses needed future work and Sect. 7 concludes our paper.

## Related work

There are numerous methods that process medical data as input to create meshes. The type of meshes generated must be taken into account when considering the target application. Meshes can be classified into two categories - structured and unstructured. While there are structured mesh generation methods that have been shown to provide accurate results within CFD simulations [15], these types of methods can sometimes fail to capture features within complex geometries [16]. Unstructured meshes can be further classified into two categories - isotropic and anisotropic. Isotropic mesh generation methods are well suited for high curvature geometries. Anisotropic meshes contain high aspect ratio elements that include directional information, potentially offering greater solution accuracy compared to isotropic meshes when used for simulations involving cerebral aneurysms and blood flow [8].

There are many methods that convert 3D images into 3D meshes. For a comprehensive overview of these methods, see [17]. Many 3D isotropic methods utilize Delaunay refinement techniques [12, 18, 19]. A challenge with Delaunay refinement, which still remains an open problem, is that almost flat tetrahedra (slivers) can survive known heuristics designed to remove them [20]. These low quality elements introduce error when processed by a solver. Additional I2M methods utilize lattice space-tree (octree) decomposition techniques [21–23] to provide meshes with adaptively-sized, high-quality elements. Some also utilize the Dual Contouring technique to improve element quality [24, 25]. There are methods which utilize parallel mesh generation techniques (with either CPUs [15, 25] or GPUs [25]). The method in [15] focuses on generating Cartesian cut-cell (structured) meshes while [25] focuses on generating isotropic tetrahedral meshes from biomolecular complexes (as opposed to 3D images).

Some methods focus on converting medical images into anisotropic meshes. Although potentially useful for blood flow simulations, the method in [26] converts a segmented image into an anisotropic mesh to then construct a high-fidelity virtual model of a fractured pelvis. The method in [19] generates high-quality adaptive hybrid meshes (anisotropic/isotropic tetrahedra for different regions and a prismatic boundary layer) for fluid-solid geometries within cardiac environments. Although the method generated high-quality hybrid meshes, the Delaunay-based isotropic elements in fluid flow regions (such as in the coronary arteries) were identified as a drawback, with anisotropy suggested as an improvement. Additionally, Delaunay tetrahedralization limits the quality of the tetrahedral core for complex biological geometries [19] and can fail to terminate for certain cases [10]. Another method was developed to model cerebral aneurysms for fluid–structure interaction simulations [8]. While the simulation results of this study demonstrated the benefit of utilizing anisotropic meshes rather than isotropic meshes, the mesh generation method is sequential, as with all the aforementioned adaptive anisotropic methods.

Our pipeline of software tools satisfies quality, fidelity, and smoothness requirements while providing near real-time performance with regards to adaptive anisotropic mesh generation. Additionally, robustness is addressed given the pipeline’s ability to process segmented images and CAD models [11]. To the best of our knowledge, there is no single method that provides all of the above. Despite the drawbacks of Delaunay-based methods, we extend PODM (given that it previously converted a segmented aneurysm image into a mesh of reasonably good fidelity and quality [10]) to not only adapt a mesh based on the curvature of the image boundary but to also approximate a user-defined sizing function to allow for adaptivity within the volume. This potentially makes the method suitable for vascular flow simulations, where the next step is to test the method with error-based sizing criteria within a simulation. While some of the aforementioned methods are capable of generating adaptive (within the volume) isotropic meshes of good quality, these methods are sequential [18, 19, 21, 22]. PODM’s parallel speculative execution model offers real-time performance even when generating up to approximately 50 million elements (in less than a minute, as seen in Sect. 4.5), and was shown to be the fastest Delaunay-based I2M method compared to other state-of-the-art software in [12]. We test both methods’ (converting segmented medical images to either isotropic or anisotropic meshes) feasibility as options to satisfy medical image-to-mesh conversion requirements when generating small-size meshes (e.g., about 1 million elements) and larger meshes (up to about 100 million elements).

## Method

We present two contributions to address the real-time performance challenge of anisotropic mesh generation within the context of medical image-to-mesh conversion.

### I2M pipeline

**Fig. 1:**
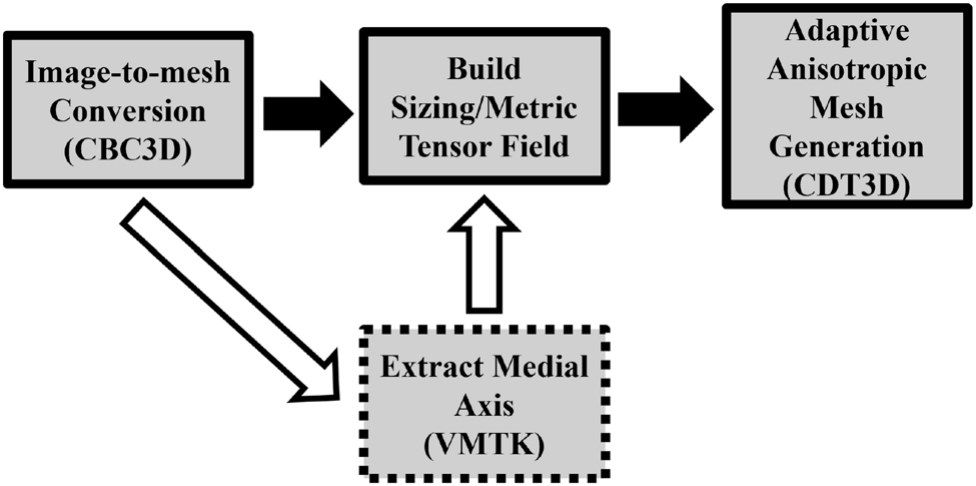
Adaptive anisotropic image-to-mesh conversion pipeline

Our anisotropic mesh generation method is integrated into a pipeline that (1) utilizes CBC3D to convert a segmented image into a high-quality and high-fidelity isotropic background mesh (for anisotropic mesh adaptation), (2) constructs an anisotropic metric tensor field, and (3) utilizes CDT3D to generate an anisotropic mesh volume using the metric tensor field. A visual overview of this pipeline of tools can be seen in Fig. 1. A script is utilized to execute this sequence of software, supplying the output of one program as input to the next. Extracting the medial axis with VMTK [27] is optional, as this technique utilized to build an anisotropic metric tensor field only serves as an example for the purpose of testing the pipeline’s robustness.

**Fig. 2:**
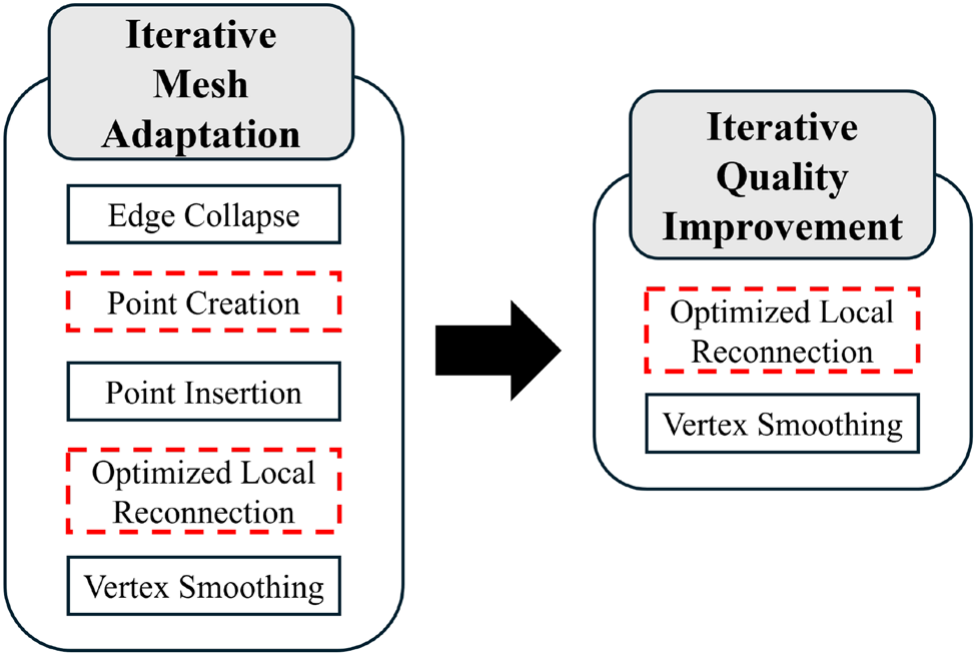
Pipeline of operations in CDT3D. Operations in red dotted lines utilize the hierarchical load balancing technique. The others are either sequential (i.e., point insertion) or utilize an OpenMP construct to exploit parallelism (i.e., edge collapse and smoothing)

CBC3D [10] converts a segmented, multi-labeled image into an isotropic, high-quality tetrahedral mesh with a surface smoothness of $C^0$ using a Body-Centered Cubic (BCC) lattice and mesh deformation technique. CBC3D is also utilized because it was previously used to discretize patient-specific medical imaging data and CAD-based stent geometries for use within CFD simulations [4]. A constructed anisotropic metric tensor field and the CBC3D volume mesh are provided as input to CDT3D. In this study, surface adaptation is disabled in CDT3D to ensure that the high-fidelity and smooth surface generated by CBC3D remains intact. CDT3D is a multicore cc-NUMA-based (shared memory) mesh generation method that exploits fine-grain parallelism at the cavity level using data decomposition. It is capable of both isotropic [28] and adaptive anisotropic [11] mesh generation. A speculative execution model is implemented, which uses atomic lock instructions to allow different data to be modified concurrently by threads while guaranteeing correctness (i.e., conformity). A lock attempts to acquire the necessary dependencies for a corresponding operation. If unable to do so, any acquired resources are released and the operation is applied on a different set of data. See [28] and [11] for more information on the design and implementation of CDT3D’s various meshing operations. CDT3D’s adaptation is organized into two phases - mesh adaptation and quality improvement. Each phase applies a particular subset of CDT3D’s operations to the mesh over numerous iterations. The mesh adaptation phase is designed to focus on modifying elements so that they conform to point spacing as required by the metric tensor field. The quality improvement phase focuses on improving element shape (i.e., improving element mean ratio, defined in Sect. 4.2). In addition to processing CAD geometries, CDT3D accepts an analytic or discrete metric field as input when performing adaptation. Figure 2 shows the sequence of CDT3D’s operations and where this paper’s contributions (the hierarchical load balancing model and optimized local reconnection operation, algorithms 1 and 2, respectively) fit into the method.

To gauge the pipeline’s robustness, our CDT3D method is tested using two different techniques for constructing a metric tensor field. For the first aneurysm case, the metric is constructed based on a technique also utilized for non-rigid registration applications intended for image-guided neurosurgery [29]. This technique focuses on capturing registration points within an adaptive mesh by constructing minimum volume bounding ellipsoids around mesh points and a specified number of their surrounding registration points. These ellipsoids have a natural mapping to a 3 $\times$ 3 positive definite matrix that can be used as a metric to guide adaptivity. See [29] for more details on this technique. To exploit parallelism, an OpenMP construct is utilized when processing the mesh points and constructing these ellipsoids. For our first test case, we utilize this technique using the points of an approximate medial axis of the generated CBC3D mesh (in place of registration points). The approximate medial axis (also called a centerline) is obtained for our first test case using VMTK [27] and can be seen in Fig. 4a of our results.

For our second aneurysm test case, we utilize streamline data of the velocity field from a CFD simulation involving blood flow executed outside of this study [30]. Because this aneurysm case was only provided to us as a surface mesh, an isotropic volume mesh is first generated using the surface mesh. The 3 closest streamline velocity points to each isotropic mesh volume vertex are used to calculate a velocity gradient tensor, which can be mapped to a 3 $\times$ 3 positive definite matrix. The magnitude (eigenvalue) of each velocity gradient tensor is used to apply a stretching factor to the positive definite matrix which ensures that elements are stretched in the dominant direction of the velocity. OpenMP is again utilized to parallelize the processing of the mesh points. The isotropic volume mesh is processed as a background mesh with the velocity-based metric tensor field to generate an anisotropic mesh.

### Hierarchical load balancing

A hierarchical load balancing technique was previously seen to enhance the performance of the I2M Delaunay-based multi-threaded software PODM, which also utilizes a speculative execution model [12] (discussed in more detail in Sect. 4.1). Consequently, we introduce a cc-NUMA-based hierarchical load balancing technique for the adaptive anisotropic parallel mesh generation software CDT3D. Before adaptation, CDT3D organizes tetrahedra into “buckets" that are all assigned to pinned threads (i.e., each thread is pinned to a CPU core) [11, 28]. When a thread finishes processing its list of buckets during adaptation, it is inserted into an idle list. Originally, after processing a bucket, a busy thread (those that still have buckets to process) would give a fraction of its buckets to the first thread in the idle list (if the list is not empty) to balance workloads. This load balancing technique was implemented for CDT3D’s more expensive (i.e., time-consuming) operations—point creation and local reconnection. It is not implemented for the edge collapse or vertex smoothing operations, as an OpenMP construct was determined to provide good parallel efficiency among various test cases in [11] for those two operations. With regards to point creation and local reconnection load balancing, CDT3D has been updated so that a busy thread will search the list of idle threads to find the pinned thread that is located within the shortest NUMA node distance among all the idle threads. cc-NUMA-based shared memory architectures organize CPU cores into NUMA nodes, where one NUMA node will consist of multiple CPU cores. When a thread creates new elements, their respective data structures are allocated in memory that is local to the NUMA node within which that pinned thread’s CPU core is located. Any CPU core (and its pinned thread) within that same NUMA node will relatively have the same memory access time to those data structures. Remote memory accesses (by CPU cores located in other NUMA nodes on the shared memory system) require greater access time. Consequently, it is ideal for busy threads to give workloads to threads that are pinned to CPU cores within the shortest NUMA node distance to reduce the number of remote memory accesses and limit the latency of such accesses during adaptation. Both the syscall^1^ and NUMA^2^ libraries are utilized to get the NUMA node id of a CPU core and the distance between two NUMA nodes (given their ids), respectively. Algorithm 1 gives an overview of CDT3D’s hierarchical load balancing. We evaluate the effectiveness of such a technique compared to CDT3D’s original load balancing method in Sect. 4.4.

**Algorithm 1:**
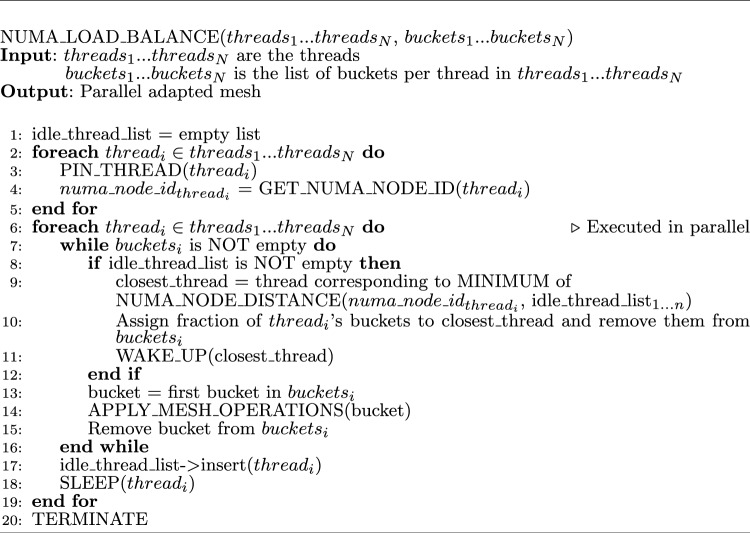
CDT3D’s hierarchical load balancing

### Optimized local reconnection

While generating good quality meshes compared to several state-of-the-art methods [14], CDT3D is simply designed to attempt to improve element quality (during its quality improvement phase) by applying mesh operations to them over numerous iterations until little to no improvement is observed or a user-defined iteration threshold is reached (not guaranteeing that all elements will be of high quality). A problem is that while the local reconnection operation (consisting of topological transformations, or flips [11, 28]) improves some elements during each iteration, it will also waste time attempting to improve other elements that were not improved successfully in a previous iteration (repeatedly processing the same elements regardless of whether or not an element or its neighbors were modified in a previous iteration). We found that on average, the operation simply repeats itself (for more than one iteration) attempting to improve approximately 6–8% of about 100 million elements generated for our second aneurysm test case. The operation has now been optimized to avoid unnecessary repetition when attempting to improve element quality. It keeps track of whether or not flips have already been attempted for an element, and recognizes that it should not re-attempt improvement if the element or its neighbors have not been modified since the last attempt (from a previous iteration). The method also recognizes if flips should be re-attempted due to unsuccessful dependency locking (when attempting to improve the element during a previous iteration) under CDT3D’s speculative execution model. Algorithm 2 shows this new functionality. The amount of time saved and the impact of this optimization can be seen in Sect. 4.5.

**Algorithm 2:**
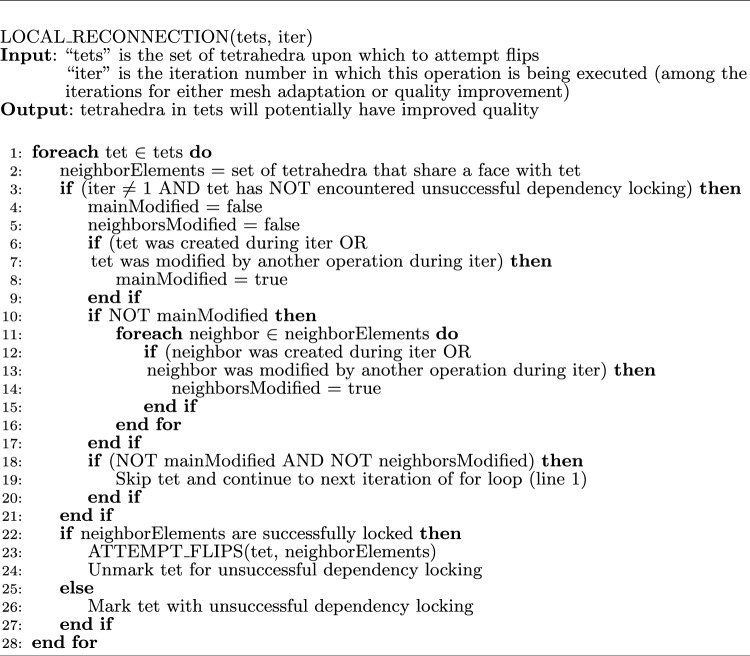
Optimized local reconnection

## Results

First, we briefly introduce PODM in Sect. 4.1 and describe our extension to its function approximation capability, which was implemented to compare its isotropic adaptation performance to CDT3D’s anisotropic adaptation. We then describe how our experiment is set up in terms of the test cases and the supercomputers utilized to test the methods in Sect. 4.2. Next, we evaluate the adaptive anisotropic I2M pipeline and adaptive isotropic I2M method in Sect. 4.3, followed by a study of the hierarchical load balancing model in Sect. 4.4 and the optimized local reconnection algorithm when generating larger meshes in Sect. 4.5.

### Parallel optimistic delaunay method (PODM)

PODM is a multicore Delaunay-based image-to-mesh conversion software that employs dynamic point insertion and removal techniques while offering quality and fidelity guarantees (referred to as PI2M in [12]). Processing a multi-labeled segmented image as input, PODM recovers the isosurface of the image object while meshing the volume concurrently. It is capable of generating millions of high quality elements in seconds while respecting the exterior and interior boundaries of tissues. To guarantee quality and fidelity, PODM adheres to specific criteria when determining if elements should be adapted (i.e., split). Introduced in [31] to specifically address nuclear femtography problems, the user can specify their own criteria through a sizing function that can be loaded as a shared library (which PODM will utilize while generating a mesh in parallel). We gauge the feasibility of utilizing PODM to satisfy I2M requirements, given its previous performance when generating good quality meshes for a segmented aneurysm image with reasonably good fidelity in [10]. The method also utilizes a tightly-coupled speculative execution model, employing carefully designed contention managers, load balancing, and synchronization techniques. Its “hierarchical work stealing" model prioritizes work load balancing among threads within the same level of hardware. For example, busy threads give work to idle threads within the same socket, and if all threads within the same socket are busy, work is given to idle threads in another socket that is located within the same blade (in the Blacklight supercomputer on which the method was tested). This model contributed to its excellent performance when generating large meshes (e.g., 1 billion elements), achieving a speedup of approximately 123 when utilizing 144 cores [12]. Consequently, we extend the method’s function approximation capability to now generate an adaptive isotropic mesh for an aneurysm case. For our first test case (the segmented image), we construct a sizing function in a similar manner as described in Sect. 3.1, based on [29]. This sizing function is utilized to determine if an element should be adapted. 3 $\times$ 3 positive definite metric tensor matrices are calculated for each of the element’s points based on their distance to the closest landmark [29] (i.e., the closest point along the medial axis). Next, the sizing function checks if any of the element’s edges have a “metric length" that is larger than a specified threshold. The metric length is a measure of how long an edge is in the space defined by the metric tensor [32]. The average of the tensors at the two endpoints of an edge is calculated to obtain an interpolated tensor for that edge. If the calculated metric length exceeds the threshold, the element is marked for refinement. Although the tensor for an edge is calculated differently than the techniques used in [33], we use this simple technique to test PODM’s ability to utilize a custom function and verify that elements are indeed adapted along the geometry’s medial axis for the first case (seen in Fig. 4d). The algorithm for this particular function can also be seen in [34].

### Experimental setup

**Fig. 3:**
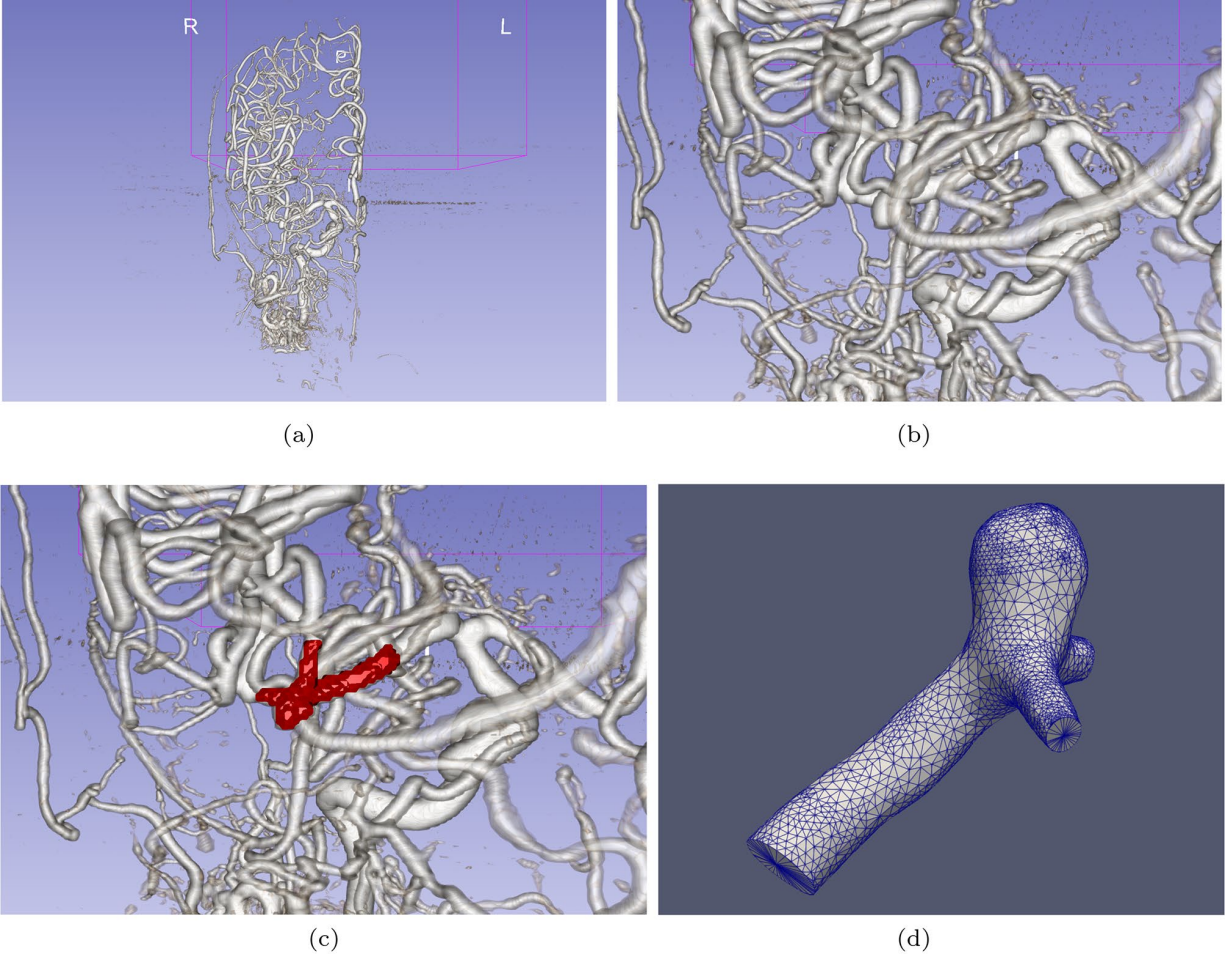
The original image of the second (middle cerebral artery bifurcation) aneurysm case is shown from an anteroposterior view. **a** Shows the vascular structure imaged by rotational angiography of the right carotid artery. **b** zooms in to the region of interest while **c** highlights the PLC obtained from the segmentation of the aneurysm. **d** Shows only the resulting PLC

**Fig. 4:**
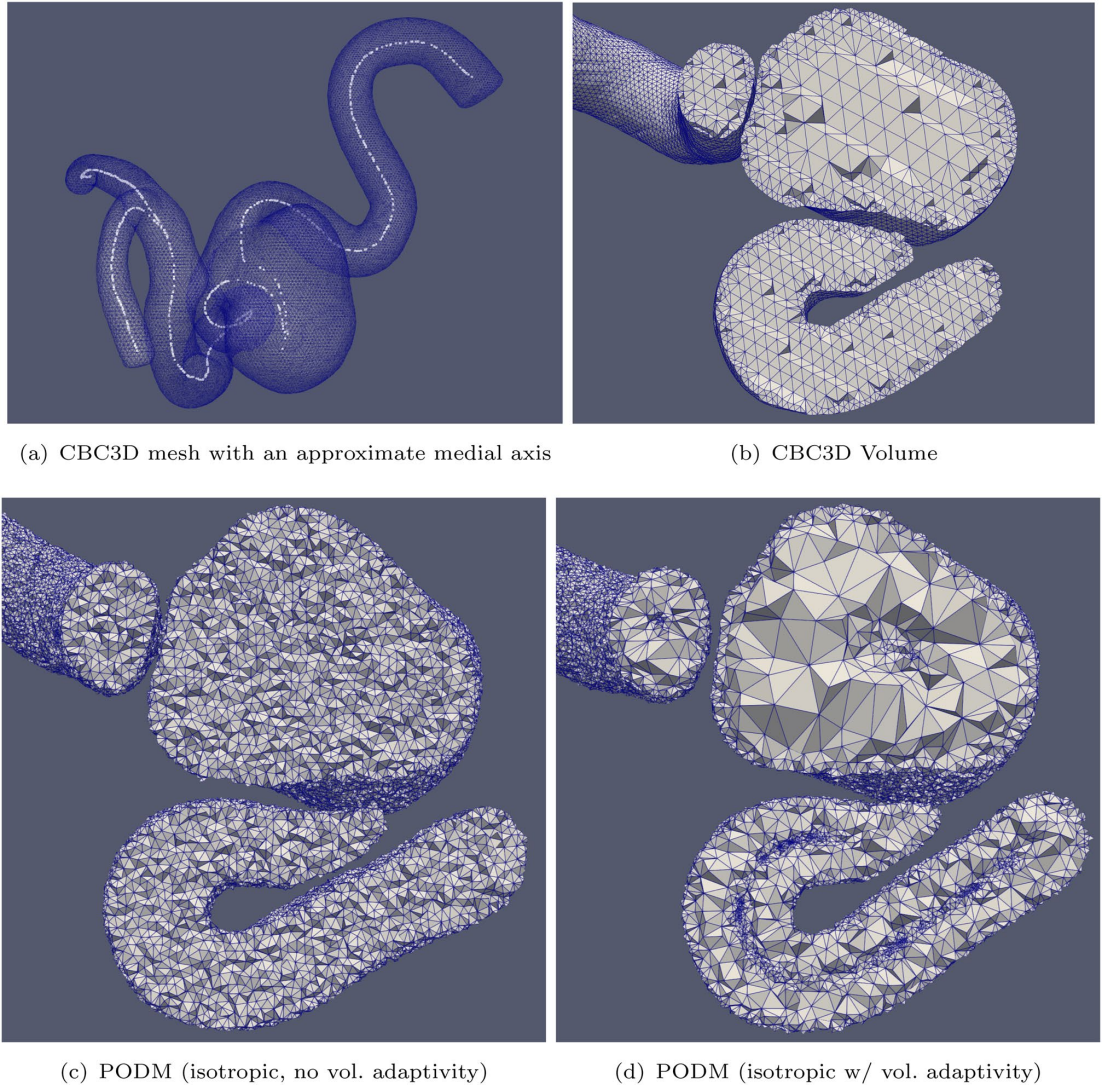
Cross sections are shown of the volume meshes generated by CBC3D and PODM for the first aneurysm case

The first aneurysm case is based on a rotational angiography scan of a carotid cavernous aneurysm with image spacing of 1.00 $\times$ 1.00 $\times$ 1.00 mm$^3$ and an image size of 512 $\times$ 512 $\times$ 508 voxels$^3$. The segmented vascular domain occupies a spatial volume characterized by an Oriented Bounding Box (OBB) with principal dimensions of 164.7 $\times$ 204.8 $\times$ 368.1 mm$^3$. The orientation of the local coordinate system for this volume is defined by the following direction vectors: $V_x$: (0.522, 0.821, 0.230), $V_y$: (0.694, $-$0.253, $-$0.674), and $V_z$: ($-$0.495, 0.511, $-$0.702). The Axis-aligned Bounding Box (AABB) has physical dimensions of 226.41 $\times$ 243.72 $\times$ 324.78 mm$^3$. Mesh generation was performed strictly within these bounds: $\times$ range: ($-$368.77, $-$142.36), y range: ($-$377.13, $-$133.41) and z range: (135.82, 460.60). The CBC3D mesh generated from this case can be seen in Fig. 4a, in addition to its approximate medial axis. The second case is a surface mesh of a middle cerebral artery bifurcation aneurysm given as a Piecewise Linear Complex (PLC) in a.stl format (seen in Fig. 3d). Figure 3 shows the vascular structure imaged by rotational angiography of the right carotid artery for the second aneurysm case, in addition to the given surface mesh obtained from a segmentation of the region of interest. Recall that we evaluate the adaptive anisotropic mesh generation method with metric tensor fields that are constructed using different techniques for each case. The full anisotropic I2M pipeline is tested with the first case. Due to the fact that the second case was provided as a PLC, the full pipeline is not necessary and PODM is not tested with this case (as PODM is designed to only process 3D images as input). Consequently, only CDT3D is used for mesh generation/adaptation for the second case (where CDT3D first generates an isotropic mesh that is then adapted using the velocity-based metric tensor field to create an anisotropic mesh). All codes were compiled using GNU GCC 11.4.1. Data were collected on three supercomputers—Purdue University’s Anvil [13, 35] and Old Dominion University’s Wahab [36] and Turing [37] supercomputers. On Anvil, we utilized a dual socket node that features two AMD EPYC 7763 CPUs @ 2.45 GHz (64 slots each) and 256 GB of memory. On Wahab, we utilized a dual socket node that features two Intel Xeon Gold 6148 CPUs @ 2.40 GHz (20 slots each) and 384 GB of memory. Finally, a dual socket node in the Turing supercomputer features two Intel Xeon E5-2698 v3 CPUs @ 2.30 GHz (16 slots each) and 128 GB of memory. For the performance evaluation, each run in the following experiments was executed three times and the results were averaged.

For the generated isotropic meshes, qualitative results are examined with respect to element dihedral angles while quality is examined with respect to metric conformity for the adapted anisotropic meshes. The goal of metric conformity is to create a unit grid, where edges are unit-length and elements are unit-volume with respect to the target metric field. A comprehensive introduction to the definition and properties of the metric tensor field is provided in [38]. The complexity *C* of a continuous metric field $\mathcal {M}$ is defined as:

1 $$\begin{aligned} C(\mathcal {M}) = \int _\Omega \sqrt{det(\mathcal {M}(x))}dx. \end{aligned}$$

Complexity on the discrete grid is computed by sampling $\mathcal {M}$ at each vertex *i* as the discrete metric field *M*,

2 $$\begin{aligned} C(M) = \sum _{i=1}^{N} \sqrt{det\left( M_i\right) }V_i, \end{aligned}$$

where $V_i$ is the volume of the Voronoi dual surrounding each node. The complexity of a grid is known to have a linear dependency with respect to the number of points and tetrahedra, shown theoretically in [38] and experimentally verified in [39, 40]. As shown in [14, 38], scaling the complexity of a metric can generate the same relative distribution of element density and shape over a uniformly refined grid compared to the original complexity. The metric tensor $M_{C_r}$ that corresponds to the target complexity $C_r$ is evaluated by [38]:

3 $$\begin{aligned} M_{C_r} = \left( \frac{C_r}{C(M)}\right) ^\frac{2}{3} M, \end{aligned}$$

where *M* is the metric tensor before scaling and C(M) is the complexity of the discrete metric before scaling. We also evaluate the performance of our parallel adaptive anisotropic mesh generation method by scaling the complexity of the metric tensor fields to generate larger meshes (up to 100 million elements).

For calculating edge length and element mean ratio of the meshes generated by CDT3D, we adopted the same definitions that appear in [33]. For two vertices *a* and *b*, an edge length in the metric $L_e$ can be evaluated using:

4 $$\begin{aligned} \begin{aligned}&L_e = {\left\{ \begin{array}{ll} \frac{L_a - L_b}{log\left( L_a/L_b\right) } & \left| L_a - L_b\right| > 0.001 \\ \frac{L_a+L_b}{2} & otherwise \end{array}\right. } \\&L_a = \left( v_e^TM_av_e\right) ^{\frac{1}{2}},L_b = \left( v_e^TM_bv_e\right) ^{\frac{1}{2}} \end{aligned} \end{aligned}$$

and an element mean ratio shape measure can be approximated in the discrete metric as:

5 $$\begin{aligned} Q_k = \frac{36}{3^{1/3}} \frac{\left( |k|\sqrt{det\left( M_{mean}\right) }\right) ^\frac{2}{3}}{\sum _{e\epsilon L} v_e^TM_{mean}v_e}, \end{aligned}$$

where *v* is a vertex of element *k* and $M_{mean}$ is the interpolated metric tensor evaluated at the centroid of element *k*. Given that optimal edges should be unit-length, edges with length above or below one are considered to be sub-optimal. The measure for mean ratio is bounded between zero and one since it is normalized by the volume of an equilateral element. One is the optimal quality for an element’s mean ratio shape.

### Quantitative and qualitative analysis of I2M pipeline

The small-size mesh results in this subsection were obtained from executing the methods on the Wahab supercomputer. Both the isotropic and anisotropic results obtained from CDT3D are compared to PODM [12] for the first carotid cavernous aneurysm case. In addition to an anisotropic metric field, an isotropic sizing function is constructed (based on the aforementioned approach in Sect. 4.1) specifically for PODM. We compare results between meshes generated by PODM when it utilizes this sizing function and when it does not (PODM’s default image-to-mesh conversion). The particular parameters used for each mesh generation method can be found in Table 1 of [34]. The first aneurysm case was also used to evaluate CBC3D in [10] (as it was compared to other isotropic I2M conversion methods from industry and academia). The same parameters utilized for CBC3D in that study are utilized in this evaluation.

**Fig. 5:**
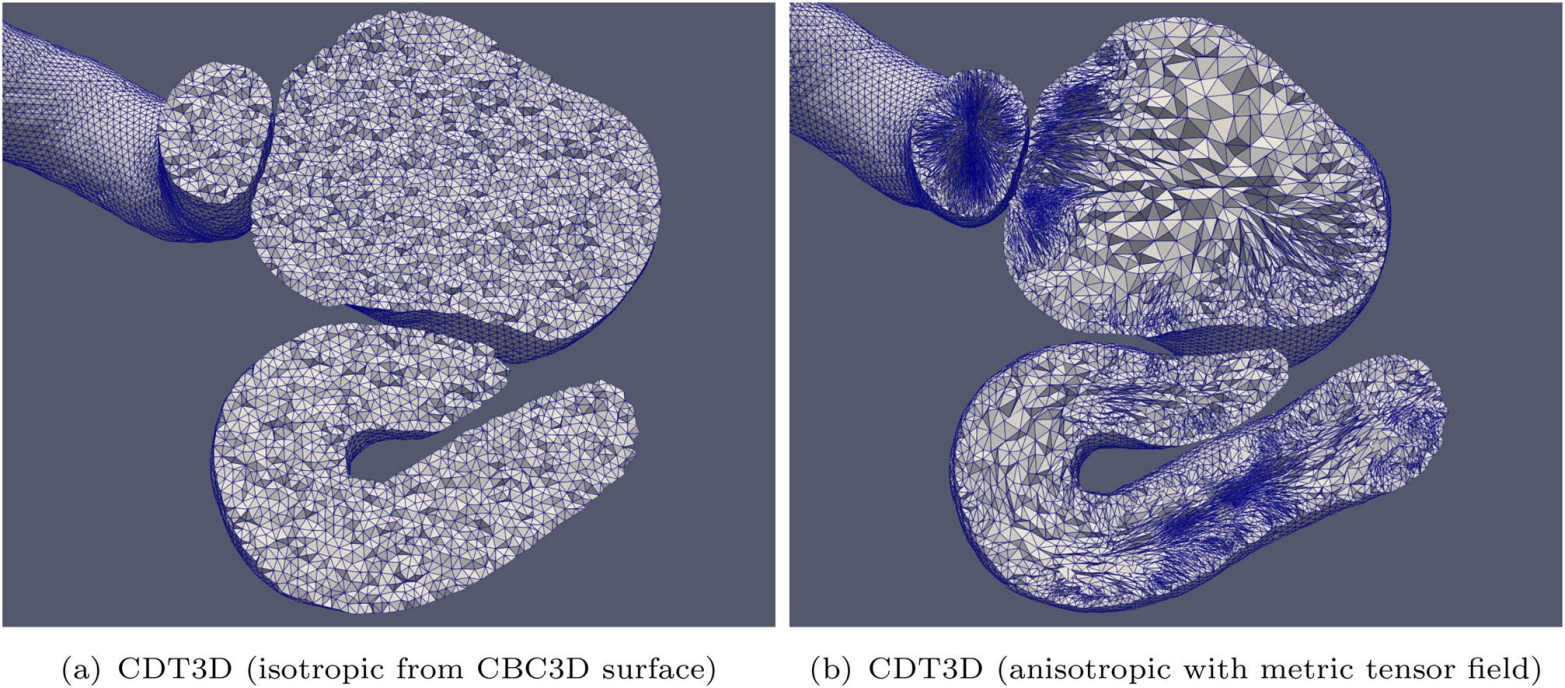
Cross sections are shown of the volume meshes generated by CDT3D for the first aneurysm case

**Fig. 6:**
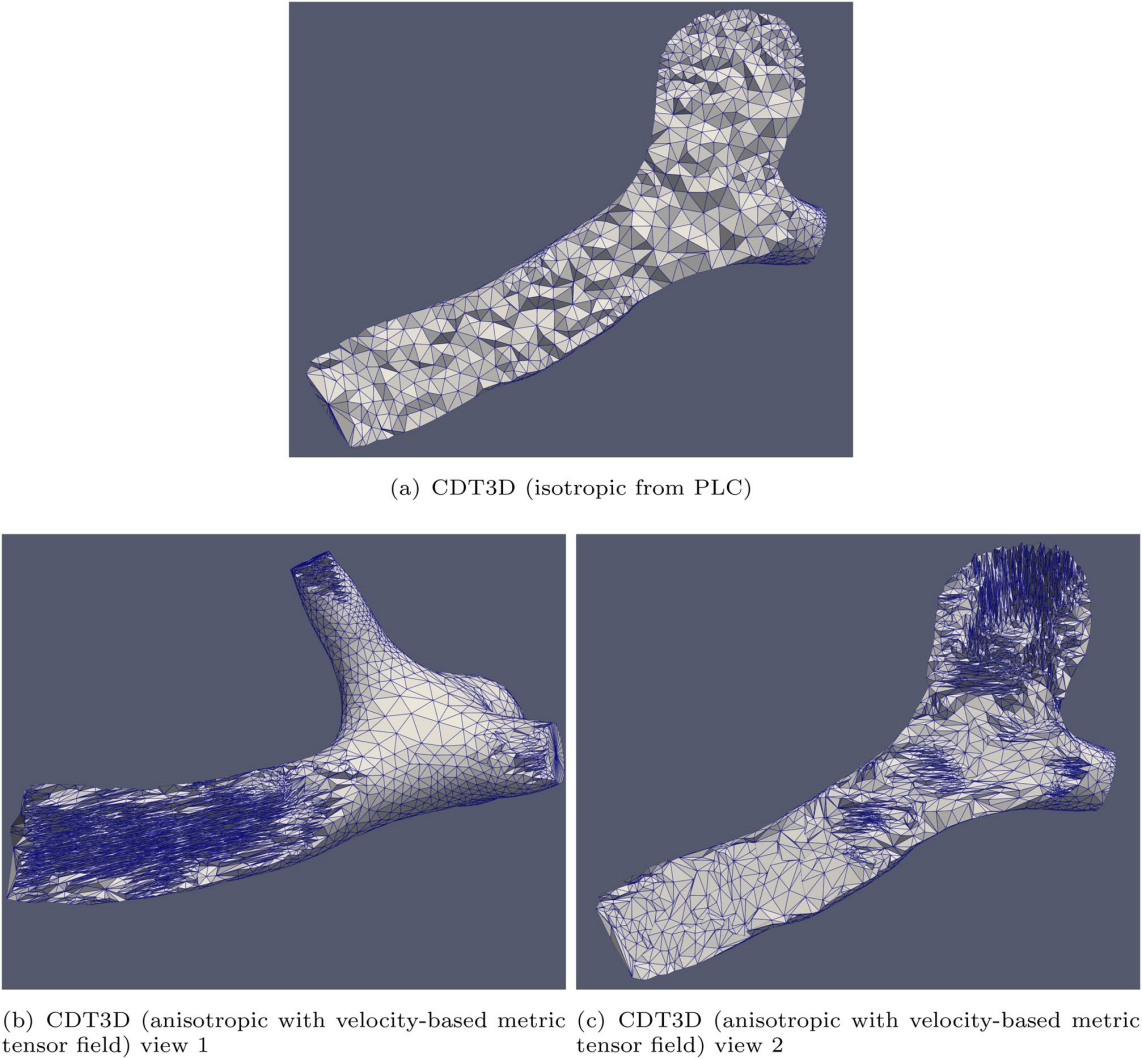
Cross sections are shown of the volume meshes generated by CDT3D for the second aneurysm case

CBC3D takes approximately 1 min to convert the segmented image of the first aneurysm into a mesh of approximately 272K tetrahedra and 63K points. Figures 4 and 5 show cross sections of the volume meshes generated by each method. Figure 6 shows cross sections of the volume meshes generated for the second aneurysm case. Table 1 shows the number of elements generated by each method for the carotid cavernous aneurysm (case 1) and the middle cerebral artery bifurcation aneurysm (case 2). Tables 2 and 3 show the runtime of each method for cases 1 and 2, respectively. They also show the mesh generation/adaptation rates of each method (measured in elements generated per second). Although it is not listed in Table 2, it should be noted that the metric tensor field is constructed in about 4 min when utilizing 40 cores. CDT3D exhibits good scalability, as its runtime is reduced to approximately 1 min and its anisotropic adaptation rate becomes about 27K elements/sec when utilizing 40 cores for the first case. For the second case, CDT3D completes anisotropic adaptation in about 49 s when utilizing 40 cores. Using the same number of cores, PODM takes about 2 s (about 260K elements/sec) to generate an isotropic mesh for the first case without utilizing the sizing function and 4 s (about 135K elements/s) when it does.

**Table 1:** Number of tetrahedra and points generated by each method when processing either the segmented image (PODM) or the CBC3D mesh converted from the segmented image of the first (carotid cavernous) aneurysm and the surface mesh of the second (middle cerebral artery bifurcation) aneurysm

Methods		Aneurysm 1	Aneurysm 2
PODM (no vol. adaptivity)	Tets	599K	–
	Points	131K	–
PODM (w/ vol. adaptivity)	Tets	644K	–
	Points	141K	–
CDT3D (iso)	Tets	631K	31K
	Points	122K	7K
CDT3D (aniso)	Tets	1.59M	80K
	Points	284K	15K

‘Tets’ is short for tetrahedra. ‘K’ means thousand and ‘M’ means million

**Table 2:** The time spent (approximately in **minutes**) and the number of elements generated per second by each method (in parentheses) for small-size meshes on the Wahab supercomputer is shown when processing either the segmented image (PODM) or the CBC3D mesh converted from the segmented image of the first (carotid cavernous) aneurysm

	CPU cores		
Methods	1	10	20
Build_Metric	191 (5K)	19 (55K)	9 (117K)
PODM (no vol. adaptivity)	0.35 (28K)	0.05 (167K)	0.04 (217K)
PODM (w/ vol. adaptivity)	1.1 (9K)	0.15 (67K)	0.1 (101K)
CDT3D (iso)	0.6 (17K)	0.3 (42K)	0.1 (78K)
CDT3D (aniso)	20 (1K)	3 (8K)	2 (14K)

Instead of elements per second, the values in parentheses for Build_Metric represent the CBC3D volume mesh points processed per second. ‘K’ means thousand

**Table 3:** The time spent (approximately in **seconds**) and the number of elements generated per second by CDT3D (in parentheses) for small-size meshes on the Wahab supercomputer is shown when processing the surface mesh of the second (middle cerebral artery bifurcation) aneurysm

	CPU Cores		
Methods	1	10	20
CDT3D (iso)	2.2 (14K)	1.73 (18K)	1.24 (25K)
CDT3D (aniso)	910 (87)	105 (761)	69 (1K)

‘K’ means thousand

Mesh fidelity is evaluated using a two-sided Hausdorff Distance (HD) metric (based on an open source implementation [41]): $\text {HD} = \max \{ \text {HD}_{\text {I}\rightarrow \text {M}}, \text {HD}_{\text {M}\rightarrow \text {I}}\}$, where $\text {HD}_{\text {I}\rightarrow \text {M}}$ is the value of the metric from the image to the mesh, and $\text {HD}_{\text {M}\rightarrow \text {I}}$ is the value of the metric from the mesh to the image. A low HD error indicates a high fidelity. Hausdorff Distance is computed between two point sets. The first point set contains the vertices located on the mesh surface and the second point set contains the voxels located on the boundaries of the segmented material. The HD for the CBC3D-generated mesh of the first aneurysm case (in Fig. 4a, b) is 4.51. The meshes generated by PODM (Fig. 4c, d) also exhibit good fidelity, as their HD are both 2.42. The fidelity of the meshes generated by CBC3D and PODM were also evaluated for the first aneurysm case in [10]. They were both compared to several highly used I2M methods, where CBC3D exhibited good fidelity while maintaining better smoothness compared to the other methods’ meshes (including PODM). The difference in the smoothness of the boundaries generated by CBC3D and PODM can also be seen in Fig. 4. Surface refinement/adaptation is disabled for CDT3D. This ensures that the high-fidelity and smooth surface generated by CBC3D remains intact.

**Fig. 7:**
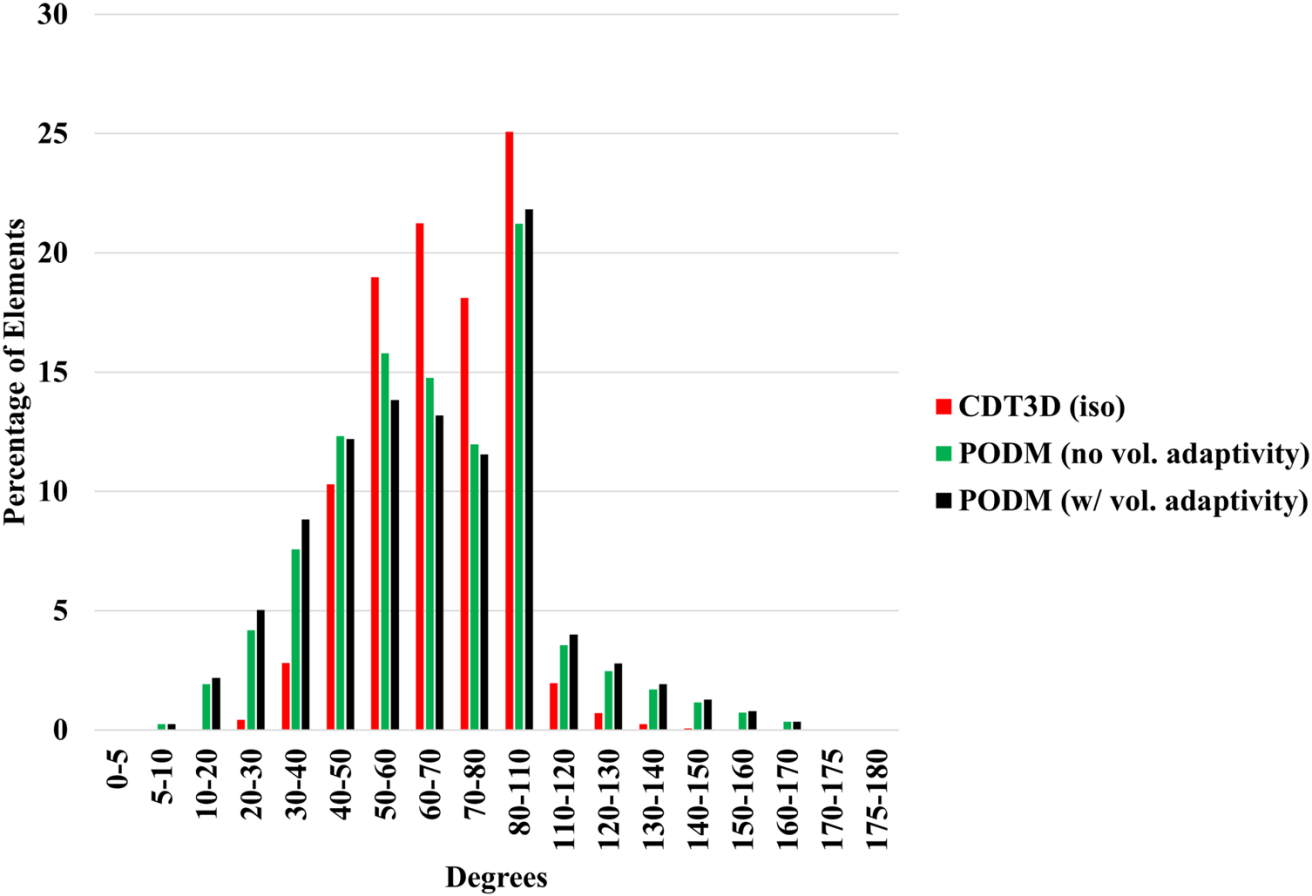
Quality statistics are shown comparing the dihedral angles of the isotropic meshes generated by CDT3D and PODM for the first (carotid cavernous) aneurysm case

**Fig. 8:**
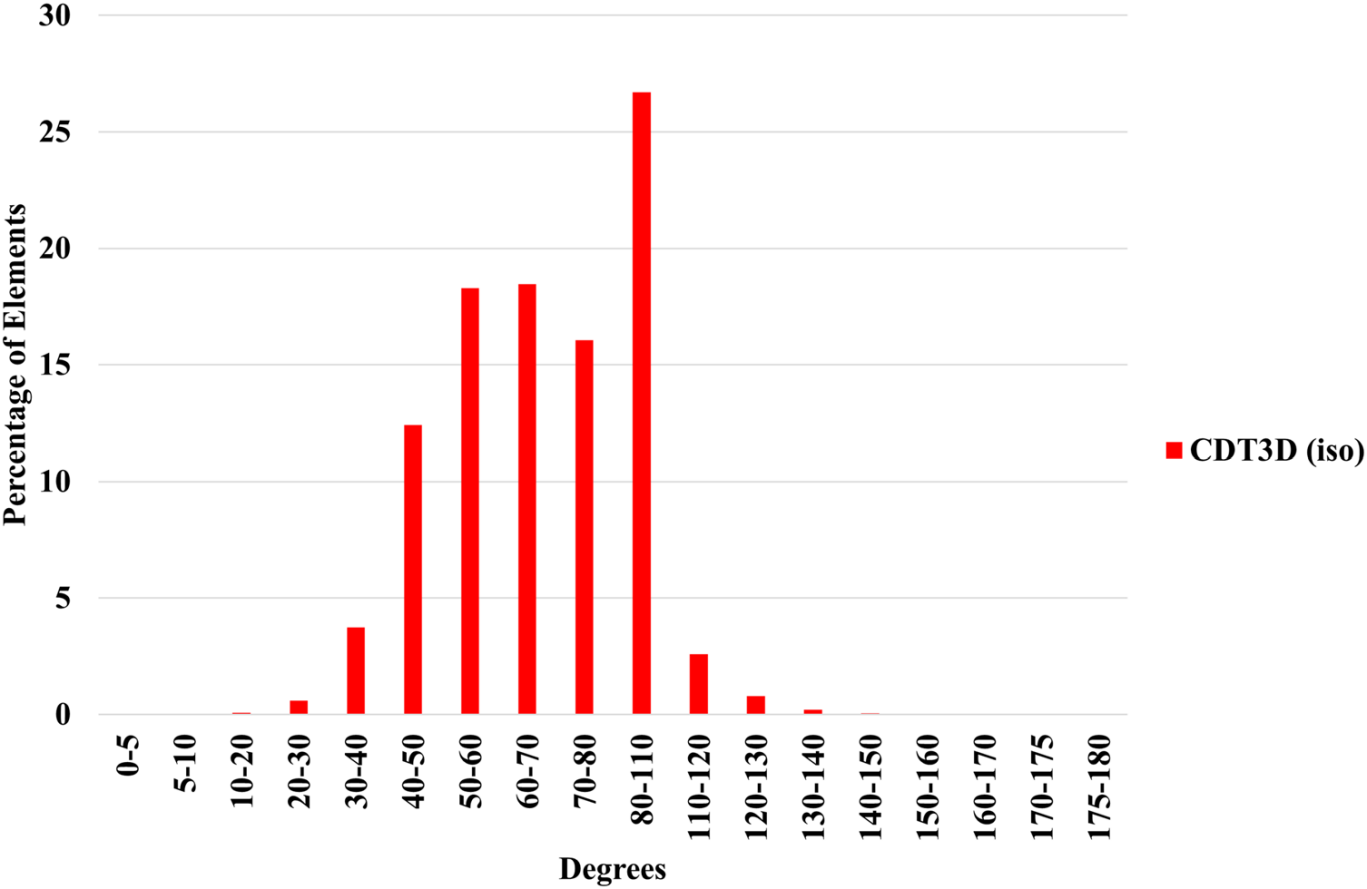
Quality statistics are shown comparing the dihedral angles of the isotropic mesh generated by CDT3D for the second (middle cerebral artery bifurcation) aneurysm case

**Fig. 9:**
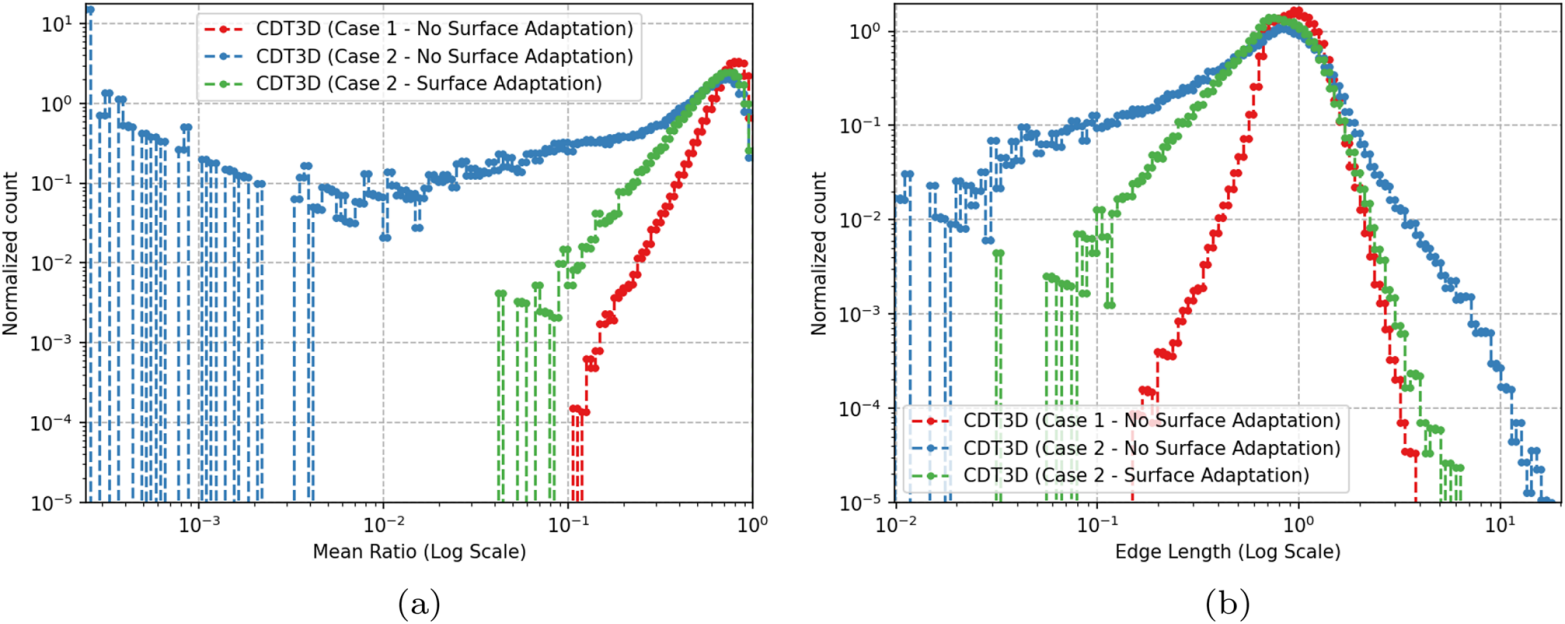
Distributions of quality statistics are shown for the mean ratio (**a**) and edge lengths (**b**) of elements within the anisotropic meshes generated by CDT3D for both aneurysm cases (in logarithmic scales)

Figure 7 compares the quality of the isotropic meshes generated by each method for case 1 and Fig. 8 shows those generated for case 2. Table 4 shows the smallest and largest dihedral angles found in the isotropic meshes generated by each method. The CDT3D meshes contain better quality compared to the PODM meshes (as they contain more elements with small and large angles compared to the CDT3D meshes). Recall that qualitative results are examined with respect to their metric conformity for the adapted anisotropic meshes. Figure 9 shows that CDT3D maintains good quality when generating an anisotropic mesh for the first case. The majority of elements have a high mean ratio and edge lengths close to 1. With regards to the second case, the anisotropic mesh generated by CDT3D exhibits poor quality when the surface of the input mesh is not adapted (containing more elements with a low mean ratio). However, when CDT3D is permitted to adapt the surface, the final mesh quality is much better. It should be noted that CDT3D converges and completes anisotropic adaptation in less time when surface adaptation is permitted (generating approximately 90K tetrahedra in about 22 s when utilizing 20 cores) as opposed to its performance when restricting the method to preserve the input surface (highlighting the constraint that the input mesh’s poor surface quality can have on the mesher’s performance if the surface must be kept frozen). The quality of the CDT3D mesh generated for the first case shows that CBC3D indeed generates a mesh of good quality when converting the first case image, given that surface adaptation is disabled for CDT3D and it still generates a final volume of good quality. Overall, the majority of elements in both anisotropic meshes generated by CDT3D for the second aneurysm case (with and without surface adaptation) have a high mean ratio and edge lengths close to 1.

**Table 4:** Smallest and largest dihedral angles in the isotropic meshes generated by each method for both aneurysm cases

Methods	Angle	Aneurysm 1	Aneurysm 2
PODM (no vol. adaptivity)	Smallest	4.71	–
	Largest	170.15	–
PODM (w/ vol. adaptivity)	Smallest	4.65	–
	Largest	170.14	–
CDT3D (iso)	Smallest	3.99	3.54
	Largest	165.25	172.93

### Hierarchical load balancing

**Fig. 10:**
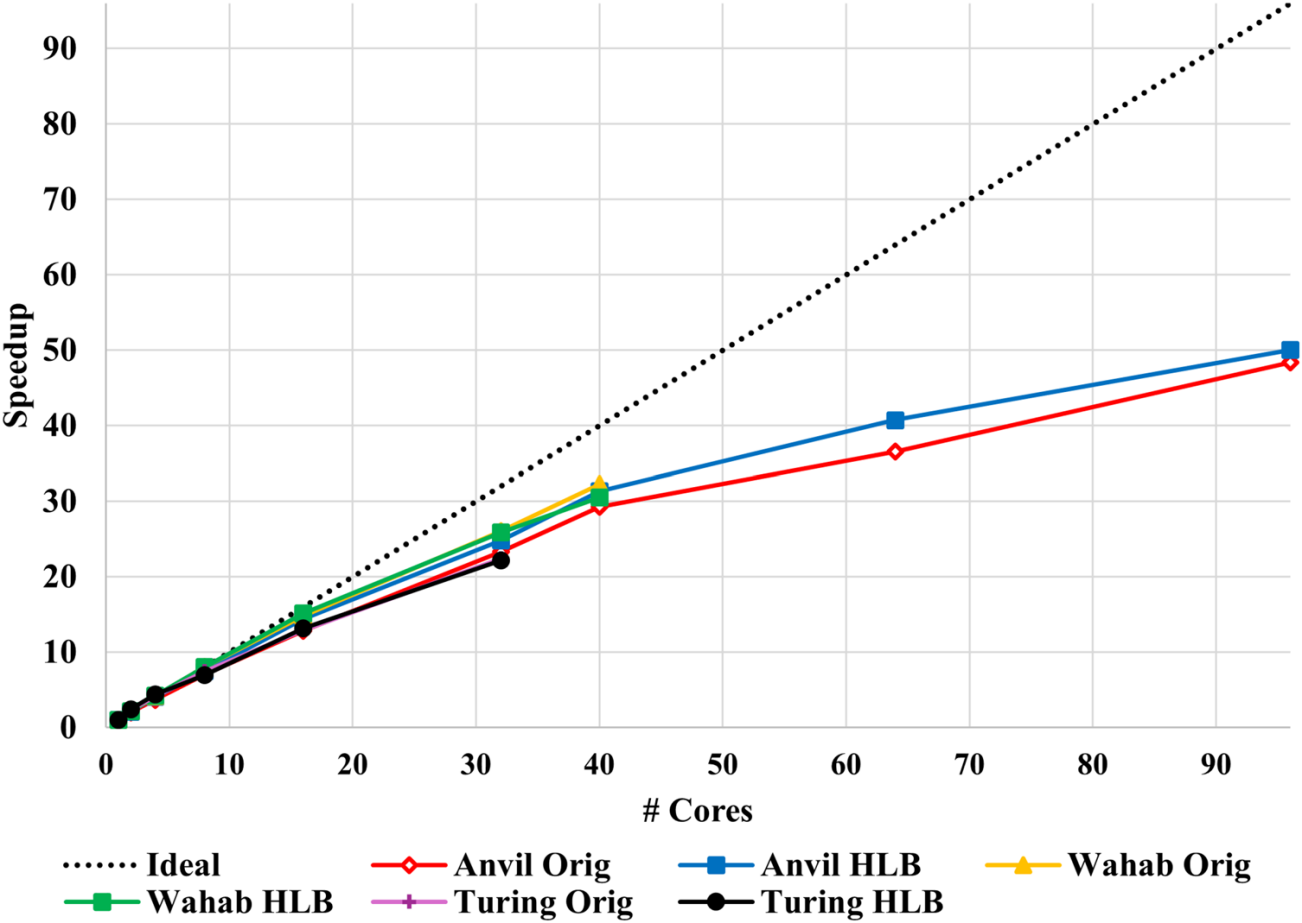
A strong scaling speedup performance comparison is given of the original (Orig) and hierarchical load balancing (HLB) versions of CDT3D when generating approximately 100 million elements on different supercomputers

We evaluate the effectiveness of the hierarchical load balancing technique using much larger test cases (i.e., more work per thread) than those seen in the previous section. Figure 10 compares the speedup achieved with and without the updated load balancing model on three supercomputers—Purdue’s Anvil, ODU’s Wahab, and ODU’s Turing supercomputers. The purpose is to gauge any potential improvements when utilizing smaller machines like the Turing supercomputer (with older specifications) versus utilizing larger machines with newer specifications (as described in Sect. 4.2). This strong scaling experiment is tested utilizing the CDT3D-generated isotropic volume mesh of the second aneurysm case from the previous section as input. The velocity-based metric tensor field is scaled from a complexity of about 20 thousand to a complexity of 50 million (generating approximately 100 million elements). We first observe that the hierarchical load balancing model gives a noticeable improvement on the Anvil supercomputer (up to about 5% additional speedup when utilizing 64 cores). On the other hand, the new model does not improve performance much on Turing and when utilizing 40 cores on Wahab. CDT3D’s original load balancing model performs slightly better than the new model on these smaller machines. A detailed analysis of the hierarchical load balancing model and its performance results is provided in Sect. 5.

### Optimized local reconnection

**Fig. 11:**
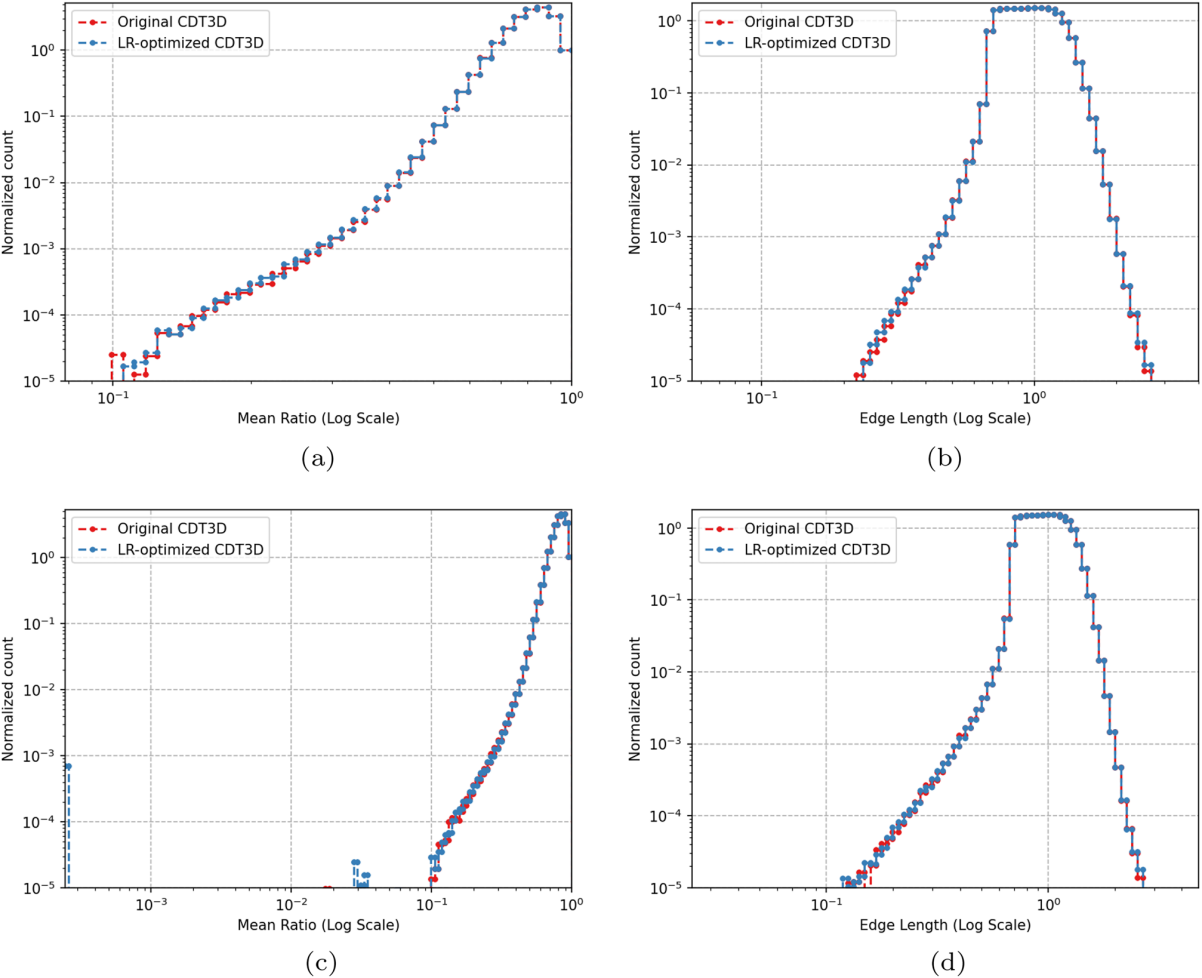
Distributions (in logarithmic scale) of quality statistics for the mean ratio, (**a**, **c**), and edge lengths, (**b**, **d**), of elements within the anisotropic meshes generated by the local reconnection-optimized CDT3D for the first (carotid cavernous) aneurysm case at 10 million complexity, (**a**, **b**), and the second (middle cerebral artery bifurcation) aneurysm case at 50 million complexity, (**c**, **d**) are shown

We gauge the performance improvement given by the optimized local reconnection (LR) algorithm in CDT3D, discussed in Sect. 3.3, by testing the updated method with both aneurysm cases on the Wahab supercomputer. We scale the complexity of the metric tensor field for the carotid cavernous aneurysm from about 180 thousand to 10 million (generating approximately 50 million elements using the CBC3D-generated volume mesh as input). We again scale the complexity of the middle cerebral artery bifurcation aneurysm case to 50 million (generating approximately 100 million elements). Table 5 shows the runtimes for the first (carotid cavernous) aneurysm case while Table 6 shows the results for the second case. Recall that there are two phases of CDT3D’s mesh generation - mesh adaptation (MA) and quality improvement (QI) [11]. The local reconnection operation is utilized during both. For both aneurysm cases, the MA local reconnection time remains about the same. This is because the mesh adaptation phase (while it does improve quality) focuses on satisfying point spacing criteria in the metric field. This means that this operation is executed over elements until little to no new points are created/inserted into the grid. On the other hand, we see a significant performance improvement for the quality improvement phase’s local reconnection operation (by about 3 times). When executed sequentially on Wahab, this saves 1 (for the first case) to 3 h (for the second case) in end-to-end time compared to the original CDT3D. When utilizing 40 cores, there is still a 3x reduction in QI local reconnection time but there isn’t as big of an improvement in end-to-end time. This is because the QI local reconnection time occupies only about 15% of end-to-end runtime in the original CDT3D for this case. This is reduced to about 5% with its optimization. The results seen specifically for the QI local reconnection time shows the performance enhancement that such an algorithm can provide for local reconnection-based methods. Figure 11 shows that the quality of the meshes generated by the local reconnection-optimized CDT3D is preserved, as they maintain comparable quality to that generated by the original CDT3D.

**Table 5:** A runtime comparison (approximately in **minutes**) of the original CDT3D and its optimized Local Reconnection version is shown when generating a mesh for the first (carotid cavernous) aneurysm at 10 million complexity on the Wahab supercomputer

Method		CPU Cores				
		1	5	10	20	40
Original CDT3D	MA Local Reconnection	232	41	21	11	7
	QI Local Reconnection	111	20	10	5	2.5
	End-to-end	501	94	49	25	15
Optimized CDT3D	MA Local Reconnection	228	40	21	11	7
	QI Local Reconnection	38	6	3	1.6	0.8
	End-to-end	421	79	42	22	13

‘MA’ means the Mesh Adaptation phase of CDT3D while ‘QI’ means the Quality Improvement phase of CDT3D

**Table 6:** A runtime comparison (approximately in **minutes**) of the original CDT3D and its optimized Local Reconnection version is shown when generating a mesh for the second (middle cerebral artery bifurcation) aneurysm at 50 million complexity on the Wahab supercomputer

Method		CPU Cores				
		1	5	10	20	40
Original CDT3D	MA Local Reconnection	524	96	52	36	18
	QI Local Reconnection	249	42	21	10	5
	End-to-end	1097	210	110	67	33
Optimized CDT3D	MA Local Reconnection	507	95	51	33	18
	QI Local Reconnection	91	13	6	3	1.6
	End-to-end	916	181	95	57	30

‘MA’ means the Mesh Adaptation phase of CDT3D while ‘QI’ means the Quality Improvement phase of CDT3D

**Fig. 12:**
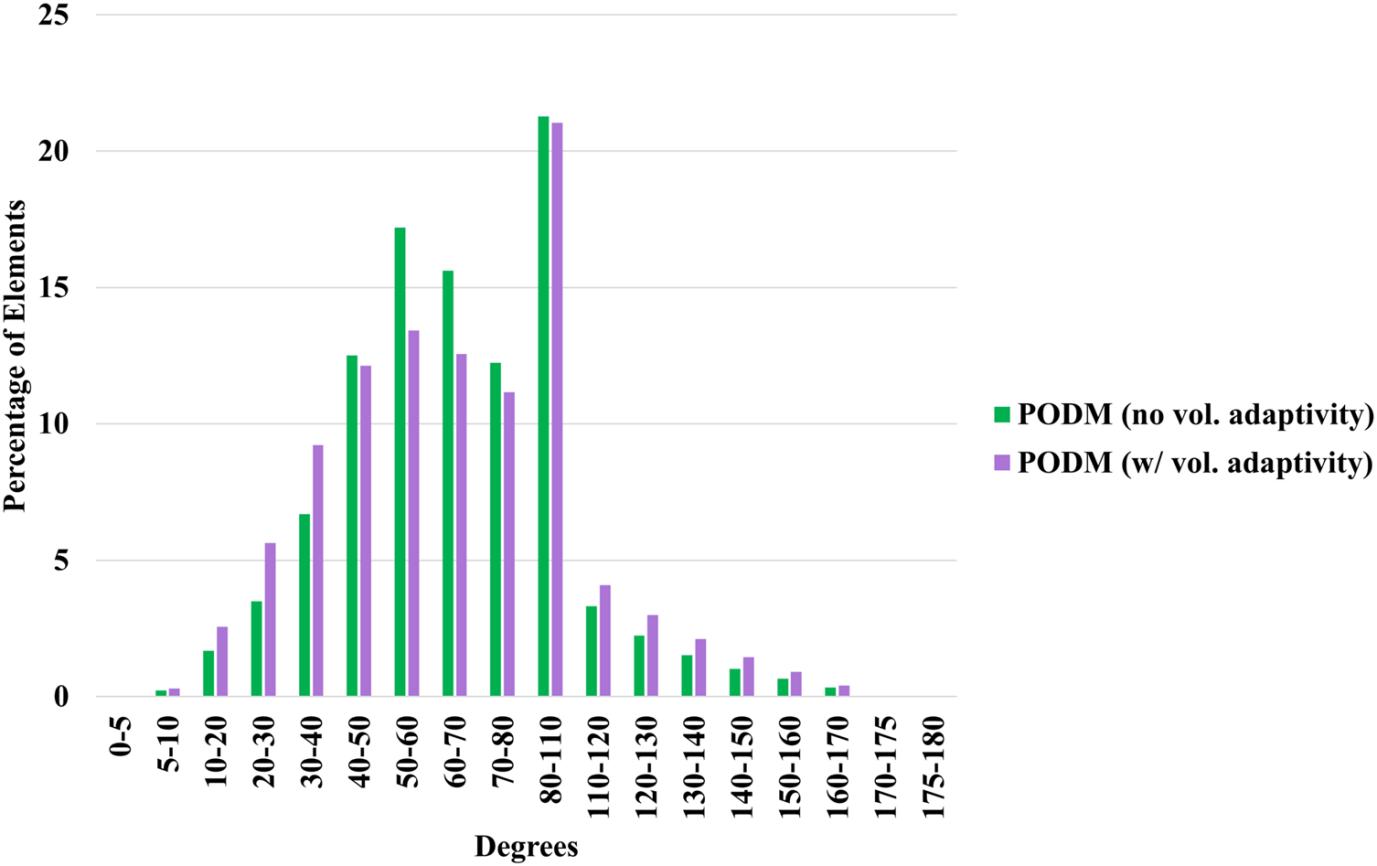
Quality statistics are shown comparing the dihedral angles of the large (approx. 50 million element) isotropic meshes generated by PODM for the first (carotid cavernous) aneurysm case

Finally, we show the runtimes when generating large meshes on the Anvil supercomputer by the optimized CDT3D (including both its hierarchical load balancing model and local reconnection optimization) and PODM’s Function Approximation (where its parameters are adjusted to generate larger meshes, see [34] for details of the method’s parameters). It should be noted that PODM’s hierarchical work stealing model is not utilized because its source code was designed and written specifically for the Pittsburgh Supercomputer Center’s Blacklight architecture. We instead utilize PODM’s random work-stealing algorithm [12] for its load balancing in this evaluation. Table 7 shows the runtimes for PODM and CDT3D when generating approximately 50 million elements for the first (carotid cavernous) aneurysm case. Table 8 shows the approximate mesh sizes generated by each method for the first case. PODM generates its isotropic meshes in less than a minute when using 96 cores while CDT3D generates its anisotropic meshes in about 5 min. PODM exhibits excellent end-user productivity by generating an adaptive mesh at a rate of about 1.17M elements/s. The optimized CDT3D exhibits an adaptation rate of about 143K elements/s for the first aneurysm case. Figure 12 shows the dihedral angle distribution of the large meshes generated by PODM. The method generates elements with the same relative distribution of element quality as with the smaller cases. Table 9 shows that the minimum and maximum angles in both meshes remain about the same. PODM maintains good quality when performing volume adaptivity to generate large meshes as well as the small ones. Table 10 shows the runtimes for CDT3D when generating approximately 100 million elements for the second (middle cerebral artery bifurcation) aneurysm case. CDT3D exhibits good scalability for both aneurysm cases. Table 11 shows the approximate mesh sizes generated by CDT3D for the second case.

**Table 7:** The time spent (approximately in **minutes**) when generating large-size meshes (about 50 million elements) for the first (carotid cavernous) aneurysm case on the Anvil supercomputer is shown

	CPU Cores							
Methods	1	2	4	8	16	32	64	96
PODM (no adaptivity)	9.6	4.8	2.4	1.3	0.7	0.4	0.3	0.2
PODM (w/ adaptivity)	50	25	13	6.6	3.4	1.8	1.1	0.7
CDT3D (Original)	294	142	75	39	21	12	8	5.9
CDT3D (Optimized)	259	122	65	35	19	10	7.8	5.5

**Table 8:** Number of tetrahedra and points generated by each method when generating large meshes (approx. 50 million elements) for the first (carotid cavernous) aneurysm case

Methods	Tets	Points
PODM (no adaptivity)	48.2M	8.6M
PODM (w/ adaptivity)	49.3M	12.2M
CDT3D (Original)	47.4M	8.1M
CDT3D (Optimized)	47.3M	8.1M

‘Tets’ is short for tetrahedra. ‘M’ means million

**Table 9:** Smallest and largest dihedral angles in the large isotropic meshes generated by PODM for the first (carotid cavernous) aneurysm case

Methods	Smallest	Largest
PODM (no vol. adaptivity)	4.35	170.29
PODM (w/ vol. adaptivity)	4.25	170.26

**Table 10:** The time spent (approximately in **minutes**) by CDT3D when generating large-size meshes for the second (middle cerebral artery bifurcation) aneurysm case at 50 million complexity on the Anvil supercomputer is shown

	CPU Cores							
Methods	1	2	4	8	16	32	64	96
CDT3D (Original)	658	325	178	93	51	28	18	13
CDT3D (Optimized)	554	265	146	81	43	24	16	12

**Table 11:** Number of tetrahedra and points generated by CDT3D when generating large meshes for the second (middle cerebral artery bifurcation) aneurysm case at 50 million complexity

Methods	Tets	Points
CDT3D (Original)	98.4M	16.6M
CDT3D (Optimized)	97.9M	16.5M

‘Tets’ is short for tetrahedra. ‘M’ means million

## Discussion

Based on the varying aneurysm mesh sizes seen in literature that are utilized to garner accurate simulation results (e.g., several thousand [8] to 200 million [9]), PODM meets the real-time requirement for both the small-size and larger-size problems in our evaluation. It is significantly faster than CDT3D due to the nature of Delaunay-based mesh generation (i.e., checking if elements satisfy the Delaunay criterion) which is not as computationally expensive as adaptation operations for anisotropic mesh generation (i.e., many more vector and matrix calculations involved in modifying elements to conform to a metric tensor field). CDT3D generates isotropic meshes with better quality than PODM but at the cost of generating more elements (for the first case compared to PODM with no volume adaptivity). As mentioned in Sect. 2 regarding Delaunay-based methods, this further showcases that local reconnection-based techniques may offer better output mesh quality than Delaunay-based techniques. Depending on the accuracy needed for numerical simulations, this tradeoff between element count/speed and quality may be acceptable, particularly when combined with CDT3D’s anisotropy. PODM’s performance in our evaluation falls in line with its excellent performance in [12], where it was shown to be the fastest Delaunay-based I2M software compared to other state-of-the-art isotropic methods. Consequently, it remains a strong candidate to satisfy medical I2M requirements if isotropic meshes are desired for numerical simulations.

Both the Build_Metric and CDT3D methods show good scalability on a single multicore node, particularly when generating an anisotropic mesh. Due to the fact that CDT3D is designed to handle much larger problems (such as that in Fig. 3 or the scaled complexity benchmarks), its performance suffers from the overhead of parallelizing adaptation when generating small meshes. Nevertheless, it also provides near real-time performance when utilizing a full multicore node (for anisotropic mesh adaptation) and when utilizing a single CPU core for isotropic mesh generation. CDT3D exhibits much faster performance when generating isotropic meshes from the PLCs of the first (CBC3D surface) and second cases. Altogether, when utilizing 40 cores on the Wahab supercomputer to generate small-size adaptive anisotropic meshes (while maintaining quality, fidelity, and smoothness) for the aneurysm cases of our evaluation, the proposed pipeline takes about 5 min for the first case and about 1 min for the second case.

Several factors can be attributed to the observations made in Sect. 4.4 about the hierarchical load balancing model and its performance. The latency introduced by remote memory accesses between NUMA nodes can still become a performance bottleneck on the Anvil supercomputer. This is because the machine is much larger (8 NUMA nodes per shared memory node of 128 cores) than Turing (2 NUMA nodes per shared memory node of 32 cores) and Wahab (4 NUMA nodes per shared memory node of 40 cores). When executing the command numactl -H at the command line on a cc-NUMA high-performance computing machine, one can see the mappings of CPU cores to NUMA nodes and more specifically, the distance between NUMA nodes. Indeed, the distance between NUMA nodes on Anvil is greater for some nodes than the farthest distance between NUMA nodes on Wahab and Turing (given that they have fewer nodes and cores than Anvil). Consequently, the hierarchical load balancing model makes an impact on a larger machine like Anvil.

To verify that the model indeed serves its purpose of reducing remote memory accesses between NUMA nodes, we also tested our method using Intel’s vtune profiler.^3^ Given that it requires special access to system files (and due to security limitations for users of the Anvil supercomputer), we only tested our method with vtune on the Wahab supercomputer (using 40 cores) when adapting the second aneurysm case at 50 million complexity. Table 12 shows several statistics given by vtune’s memory access analysis of both versions of CDT3D. The local and remote access counts refer to the total local and remote accesses among the two sockets (recall that there are two sockets in a single shared memory node in these machines). The hierarchical load balancing model indeed reduces the number of remote memory accesses by almost 50%.

**Table 12:** The approximate memory accesses of the original CDT3D’s load balancing model vs. the hierarchical load balancing (HLB) model is shown—‘M’ means million

	Original	HLB
Local Memory Accesses	11.4M	12.6M
Remote Memory Accesses	50.7M	26.4M
Remote Cache Accesses	46.4M	31.5M

This model is however slower than the original load balancing model in terms of time complexity. It must search the idle thread list and find the thread with the shortest NUMA node distance every time a busy thread attempts to give work to another. The worst case time complexity for each thread (based on algorithm 1) is $O(t\log (b))$ where t is the number of idle threads and b is the number of buckets assigned to this particular thread (as opposed to *O*(1) for the original model that simply gives work to the first available idle thread). Coupled with the fact that there are few NUMA nodes on the Wahab machine (and consequently, a less expensive distance between its 4 NUMA nodes compared to Anvil’s 8 NUMA nodes), the hierarchical model barely offers improvement in terms of speedup on Wahab. While offering an improvement on Anvil, it should be noted that the hierarchical load balancing model does not offer as much an improvement as seen in its implementation from [12]. Simply put, this is attributed to the difference in the newer specifications of Anvil as opposed to the Pittsburgh Supercomputing Center’s Blacklight machine utilized in [12]. Some of these differences include (1) DDR4 memory supported by Anvil (6 memory channels per CPU, offering higher memory bandwidth and lower latency) as opposed to the Blacklight’s DDR3 memory (4 memory channels per CPU) and (2) the Ultra Path Interconnect (UPI) links used by Anvil (up to 3 per CPU, 10.4 GT/s) compared to Blacklight’s QuickPath Interconnect (QPI) links (up to 4 per CPU but at 6.4 GT/s). The newer interconnect links reduce inter-socket latency. PODM was tested when using up to 176 cores in [12]. Remote memory accesses were more frequent and expensive on the Blacklight’s architecture of multiple blades with 2 sockets per blade (1 NUMA node per socket) and 8 cores per socket (compared to an Anvil node with 2 sockets, 4 NUMA nodes per socket, and 64 cores per socket). Overall, CDT3D’s hierarchical load balancing model offers improvement in terms of memory access and runtime on larger machines, but this improvement is minimized on a newer, single shared memory node given its advancements in architecture.

## Future work

Remote memory access latencies were obtained by running Intel’s version of the lmbench benchmark program^4^ on a node within each of the supercomputers of our evaluation. We observed that latency increases as the size of a shared memory node increases (i.e., the number of cores). When transitioning from an Anvil shared memory node to Anvil’s distributed memory environment, average message latency between NUMA nodes increases from about 190 nanoseconds to 900 nanoseconds between distributed memory nodes while bandwidth significantly decreases (memory bandwidth of about 3244 Gbps within a single node vs. the network interconnect bandwidth of 100 Gbps between distributed nodes). Bandwidth specifications are available (on their respective websites) for the CPUs utilized within Turing [42], Wahab [43, 44], and Anvil [45, 46]. Given that our hierarchical load balancing model grants additional speedup even when executed on a shared memory node (i.e., Anvil) that has low latency and high bandwidth, an even greater speedup would likely be achieved if the model was utilized in a distributed memory setting where limiting the distance of data migration becomes paramount due to the much higher latency and lower network interconnect bandwidth in distributed memory. Although focused on generating structured hexahedral meshes for explosion shock wave simulations, such a model is implemented in [47] using MPI-3 Remote Memory Access to create a virtual window for shared memory data transfer between distributed memory nodes. Although overhead increases as the number of nodes utilized increases (due to implicit global synchronization caused by shared variables between nodes), the model indeed reduces average communication latency (by prioritizing local memory accesses) and improves the parallel efficiency of the method compared to when it utilizes traditional memory access techniques. This virtual window for shared memory access between distributed nodes could potentially be applied to anisotropic meshing; however, this is outside the scope of this paper and will be addressed in future work.

Given its performance when adapting the aneurysm geometries at scaled complexities, we predict that CDT3D would be well suited for processing a geometry generated from an image of a large vascular structure (such as in Fig. 3) or when processing geometries of intrasaccular stents (where meshes of a few hundred million elements may be necessary to accurately model the stents’ affect on an artery’s hemodynamics [9]). This will be explored in future work. It should be noted that a routine within CDT3D, which attempts to remove boundary slivers [28], was disabled for the scaled second aneurysm case. This likely contributed to the low quality elements seen in Fig. 11c

The mesh deformation scheme employed by CBC3D in our I2M pipeline produces meshes with a smoothness of $C^0$. This smoothness is sufficient for aneurysm geometries such as the carotid cavernous aneurysm case in our evaluation [10]; however, future work is required to improve the smoothness of geometries reconstructed from micro-CT images (i.e., some stent geometries). Our approach must be updated to provide a smoothness of $C^1$.

Eventually, CBC3D and CDT3D should be integrated into a single software. It should be noted that conversions are required between the input and output files of the methods utilized (including VMTK when constructing the metric tensor field for the first case). For example, these methods use the VTK library [48]. Because they were each developed at different time periods, one method produces output utilizing a newer version of the library while another (which utilizes an older version) needs to read this as input. Although the conversion times for the small test cases in our evaluation are negligible, this discrepancy between the methods will likely negatively impact performance when the pipeline is tested on large cases. Integrating these methods into a single software will not only eliminate this problem, but will remove the complexity of compiling all codes separately and running them one after the other in the pipeline. Rather than separating image-to-mesh surface adaptation from anisotropic volume generation (and freezing the surface during this step), it would be more effective to perform both simultaneously when converting the segmented image (similar to PODM’s approach [12]). The inability to maintain high quality in the mesh generated for the second case when CDT3D’s surface adaptation was disabled (Fig. 9) reinforces this need. The I2M functionality provided by CBC3D must also be parallelized (or replaced with another parallel method) in order to maximize potential performance, especially for large cases. Additionally, it would be beneficial to integrate a parallel boundary layer generation technique into the pipeline (or integrate into CDT3D). Boundary layer meshes are useful for hemodynamics simulations when calculating metrics such as pressure or Wall Shear Stress (WSS) caused by blood flow [2, 19]. This will be explored in future work.

Most importantly, the next step is to integrate the anisotropic pipeline (with CDT3D) and PODM into a medical numerical simulation. Such a study will focus on comparing the accuracy and error of critical simulation metrics (such as velocity fields) to determine if the PODM-generated isotropic meshes are sufficient for clinical-grade predictions, or if the CDT3D-generated anisotropic meshes are necessary to accurately predict an aneurysm’s potential rupture. We will also measure the performance of each method within the context of the simulation’s end-to-end execution time to validate their near real-time performance capabilities. This future work is essential to confirm the end-user productivity of these approaches.

## Conclusion

Two performance optimization techniques are presented for a mesh adaptation method that is designed to help streamline the discretization of complex vascular geometries within the numerical modeling process. This method is integrated into a pipeline with another tool to generate an adaptive anisotropic mesh from a segmented image of a carotid cavernous aneurysm, providing near real-time performance, good quality, fidelity, smoothness and robustness. The adaptive anisotropic mesh generation method, CDT3D, is also tested using a surface mesh of a middle cerebral artery bifurcation aneurysm. CDT3D leverages a hierarchical load balancing model while also using an optimized local reconnection algorithm that is three times faster than its previous implementation from past studies [11]. We compare this anisotropic method to a Delaunay-based software, PODM, that generates adaptive isotropic meshes while utilizing a new user-defined sizing function, also providing real-time performance, good quality, and fidelity. When utilizing 96 CPU cores on a single, multicore node on Purdue University’s Anvil supercomputer [13], PODM generates about 50 million elements in less than a minute while the adaptive anisotropic method generates approximately the same amount in about 5 min. Such performance encourages an investment into a multi-core, shared memory machine that can potentially be utilized within a clinical setting (e.g., a single AMD processor with up to 96 CPU cores can cost about $10K). Alternatively, end users may benefit from leveraging the concurrency offered by a virtual machine (such as Google’s Compute Engine [49]) when integrating the presented methods into their numerical simulation workflows.

Both techniques (converting medical images to either isotropic or anisotropic meshes) are presented as feasible options for end users, depending on the simulation and accuracy (i.e., tradeoff between speed and the mesh type) needed. For example, isotropic meshes of cerebral vasculature have been utilized within hemodynamic simulations to accurately predict flow conditions of stents post-deployment [9]. This study notes the expensive computational cost of generating meshes with up to 200 million elements, which could potentially be generated quickly using a method like PODM (as this method generates about 1.17M elements/sec when executed with 96 CPU cores). On the other hand, the study in [8] shows that anisotropic meshes may offer more accurate results when including directional information (important for metrics like blood flow velocity). Just as the parallel anisotropic adaptation procedure (CDT3D) was tested within aerospace CFD simulations using CAD models [11], the method is expected to provide accurate results for medical numerical simulations involving CAD-based stent models in near real-time when executed on large multicore cc-NUMA (shared memory) architectures (capable of adapting about 143K elements/sec with 96 CPU cores). Having shown the feasibility of these approaches with regards to meeting medical image-to-mesh conversion requirements, the next step is to test both methods’ integration into a medical numerical simulation (while utilizing an error-based sizing function for PODM) to validate the end-user productivity of these approaches.

## Data Availability

No datasets were generated or analysed during the current study.

## Footnotes

1 https://man7.org/linux/man-pages/man2/syscall.2.html.

2 https://man7.org/linux/man-pages/man3/numa.3.html.

3 https://www.intel.com/content/www/us/en/developer/tools/oneapi/vtune-profiler.html.

4 https://github.com/intel/lmbench.
